# Typical Fluorescent Sensors Exploiting Molecularly Imprinted Hydrogels for Environmentally and Medicinally Important Analytes Detection

**DOI:** 10.3390/gels7020067

**Published:** 2021-06-08

**Authors:** Lihua Zou, Rong Ding, Xiaolei Li, Haohan Miao, Jingjing Xu, Guoqing Pan

**Affiliations:** 1 Center for Molecular Recognition and Biosensing, School of Life Sciences, Shanghai University, Shanghai 200444, China; 18201738579@163.com; 2 Sino-European School of Technology of Shanghai University, Shanghai University, Shanghai 200444, China; Rongding_2021@163.com (R.D.); xiaolei_li2020@163.com (X.L.); 3 Institute for Advanced Materials, School of Materials Science and Engineering, Jiangsu University, Zhenjiang 212013, China; haohan_miao@163.com

**Keywords:** molecularly imprinted polymeric hydrogels, synthetic receptors, zearalenone, glucuronic acid, fluorescent sensors

## Abstract

In this work, two typical fluorescent sensors were generated by exploiting molecularly imprinted polymeric hydrogels (MIPGs) for zearalenone (ZON) and glucuronic acid (GA) detection, via the analyte’s self-fluorescence property and receptor’s fluorescence effect, respectively. Though significant advances have been achieved on MIPG-fluorescent sensors endowed with superior stability over natural receptor-sensors, there is an increasing demand for developing sensing devices with cost-effective, easy-to-use, portable advantages in terms of commercialization. Zooming in on the commercial potential of MIPG-fluorescent sensors, the MIPG_ZON is synthesized using zearalanone (an analogue of ZON) as template, which exhibits good detection performance even in corn samples with a limit of detection of 1.6 μM. In parallel, fluorescein-incorporated MIPG_GA is obtained and directly used for cancer cell imaging, with significant specificity and selectivity. Last but not least, our consolidated application results unfold new opportunities for MIPG-fluorescent sensors for environmentally and medicinally important analytes detection.

## 1. Introduction

Due to the tight correlation between human health and environmental pollution that results in various diseases, environmental monitoring of pollutants and biomedical diagnosis of biomarkers are global concerns [1,2]. Hence, there is an increasing demand for developing sensors for the selective detection of various targets, such as abused pesticides and overproduced mycotoxins in food samples [3,4,5,6], as well as biomarkers and tumor cells that represent related diseases [7,8,9,10]. Moreover, the obtained sensors should be fabricated cost-effectively and capable of rapid, sensitive, portable applications. Traditionally, a natural receptor, such as enzymes, antibodies, DNA, and cells are used to react with the analyte of interest. A significant advancement has been achieved in sensors development, especially pertaining to routine procedures based on these biomolecules [11]. Despite their excellent specificity, these biosensors suffer from reagent stability and availability, high cost and cumbersome analysis procedures, etc. [12]. In this context, researchers eagerly seek synthetic tailor-made receptors capable of selectively recognizing and binding target molecules with good affinity, while being chemically and thermally stable, easy to prepare, and reusable [13]. One of the most promising strategies to create such synthetic receptors is molecular imprinting technology (MIT), which is based on the co-polymerization of functional monomers with the crosslinking agent in the presence of the template (the target molecule or a derivative thereof). After removal of the template which leaves cavities with a size, shape, and functionality complementary to the template, a tailor-made receptor called molecularly imprinted polymer (MIP) is synthesized [14,15].

Owning to the manufacture advances, the MIT has been used for a wide range of target molecules, from small molecules to macromolecules and even larger entities [16,17]. The synthesized MIP can be widely used in analyte purification, biosensing, and bioimaging, as well as drug delivery and cancer therapy [18,19,20,21,22], due to its antibody-like affinity, significant selectivity, and excellent biocompatibility [17]. However, the MIP advances towards industrial use very slowly, and even the most promising MIP-based sensors are not widely used in the industry, mainly due to the inconvenience in manufacturing and real-world use [23]. Therefore, the commercialization of MIP-based sensors relies on the simplified synthesis process and portable property for the improvement of consolidated applications in various scenarios [24].

**Scheme 1:**
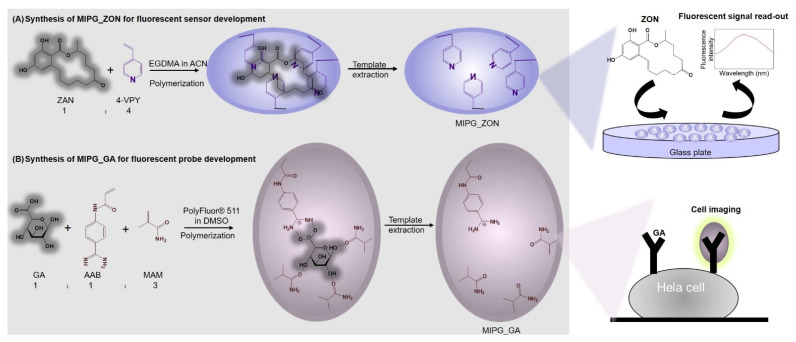
Schematic illustration of molecular imprinting technology for two typical fluorescent sensors development. (**A**) The molecular imprinting process for the synthesis of MIPG_ZON, which can be pasted on the glass substrate in order to work as a sensor for ZON detection by reading out the fluorescence signal of ZON. (**B**) The molecular imprinting process for the synthesis of fluoresce-incorporated MIPG_GA as a fluorescent probe which can be directly used for cancer cell imaging.

Aiming at developing convenient MIP-based sensors towards commercial applications in the future, two typical fluorescent sensors were generated, possessing the sensing mechanism either via the analyte’s self-fluorescence property or receptor’s fluorescence effect, respectively (Scheme 1). Specifically, a molecularly imprinted polymeric hydrogel (MIPG) was synthesized for zearalenone (ZON), a mycotoxin produced by several species of Fusarium molds to contaminate cereals, in particular corn, barley, oats, wheat, and sorghum [25], causing toxic effects in humans and animals, including nephrotoxic, neurotoxic, carcinogenic, estrogenic, and immunosuppressive effects [26]. Specifically, MIPG_ZON was developed using an optimized amount of 4-vinylpyridine (4-VPY) as a functional monomer and ethylene glycol dimethacrylate (EGDMA) as a cross-linking monomer against the zearalanone (ZAN, an analogue of ZON but without a double bond, which may interfere with the polymerization process). Afterwards, the synthesized MIPG_ZON was grafted on the glass plate to detect the ZON in corn juice. In parallel, a fluorescein-incorporated MIPG was generated for the direct recognition of glucuronic acid (GA), a small monosaccharide as the epitope of hyaluronic acid, the biomarker of cancerous or infectious cells on the cell surface [27,28,29]. The obtained MIPG_GA was fluorescein dimethacrylate (PolyFluor^®^ 511) cross-linked using two functional monomers, (4-acrylamidophenyl)(amino)methaniminium acetate (AAB) that interacts with the carboxylic group of GA with high affinity, and methacrylamide (MAM) which provides hydrogen bonds to GA. The final application of MIP_GA in cancer cell imaging showed a great potential of our MIP as a fluorescent probe for biomedical analysis.

## 2. Results and Discussion

### 2.1. Characterization of Synthesized MIPGs_ZON

Three MIPGs_ZON using the functional monomer 4-VPY at different proportions were synthesized (Table 1), with the expectation that the “π–π” interaction between ZON and the monomer would lead to strong affinity. According to our preliminary study (Figure S3), the double bond in the chemical structure of ZON interferes with the imprinting process, which brought no difference between MIPG and NIPG. To address this problem, an analogue ZAN was used as a template for MIPGs_ZON synthesis. The hydrodynamic diameter of the ZAN imprinted polymers was analyzed by the dynamic light scattering (DLS) on a Zeta-sizer NanoZS (results are summarized in Table 1), which shows a very homogenous size for each MIPG_ZON.

### 2.2. Equilibrium Binding Studies on MIPGs_ZON

**Figure 1:**
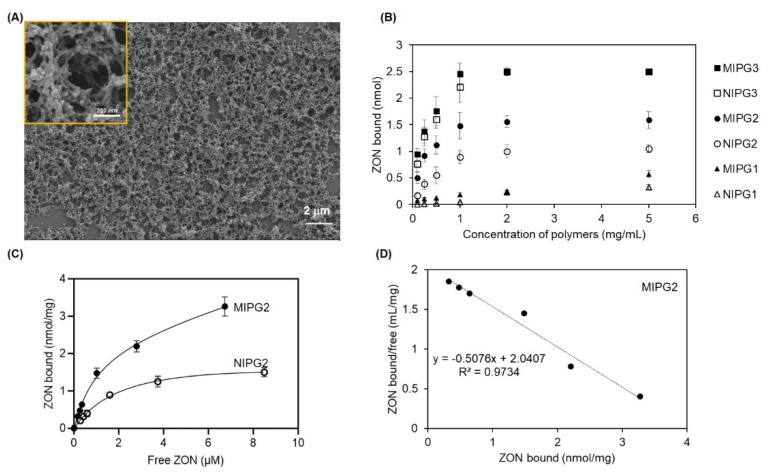
(**A**) SEM image of the MIPGs_ZON. (**B**) Equilibrium binding isotherms for 2.5 μM ZON on MIPGs_ZON at concentrations ranging from 0.1 to 5 mg/mL in 1 mL ACN. Data are means from three independent experiments. The error bars represent standard deviations. (**C**) Determination of the binding capacity of 1 mg MIPG2 and NIPG2 towards ZON varying from 0.5 to 10 μM in 1 mL ACN, respectively. Data are means from three independent experiments. The error bars represent standard deviations. (**D**) Corresponding Scatchard plot of MIPG2.

In Figure 1A, our hydrogel is a dense three-dimensional fiber network as observed under the scanning electron microscope (SEM). The relevant component analysis was carried out by a Fourier transform infrared spectrometer (FTIR, Figure S4). By magnifying the SEM image, the thickness of the fibers is found to be at the nanometer level. Figure 1B represents the binding behavior of the three MIPGs_ZON ranging from 0.1 to 5 mg/mL towards 2.5 μM ZON in ACN, indicating that the ratio ZAN: 4-VPY: EGDMA of 1:4:20 corresponding to MIPG2 gave the best binding specificity, especially for low polymer concentrations where 0.1 mg of MIP can bind to 0.5 nmol of ZON, leading to an imprinting factor of 3.0. Afterwards, 1 mg of MIPG2 and NIPG2 were incubated overnight with ZON at concentrations varying from 0.5 to 10 μM in 1 mL ACN. As shown in Figure 1C, the Langmuir-type isotherm equation was used for plotting ZON bound versus free in case of MIPG2 for the specific binding analysis, in which the binding capacity B_max_ was found to be 5.8 nmol/mg, and the dissociation constant K_d_ was 7.9 μM with R^2^ = 0.99. At the same time, the Freundlich model was used in NIPG2 cases for the non-specific adsorption analysis, it was found that B_max_ = 4.3 nmol/mg and K_d_ = 7.1 μM with R^2^ = 0.98. Therefore, MIPG2 presents higher binding capacity while a similar affinity towards ZON, with respect to NIPG2. As shown in Figure 1D, the corresponding Scatchard plot, ZON bound/free versus ZON bound is represented by a straight line, indicating a homogeneous MIPG2. The variables N and K_a_ in the Scatchard equation (B/F = K_a_N–K_a_B) correspond to the total number of binding sites and the association constant, which were found to be ~ 5.1 × 10^5^ M^−1^ and 4.0 nmol/mg, respectively.

### 2.3. Application of MIPGs_ZON-Based Fluorescent Sensor for Real Sample Tests

**Figure 2:**
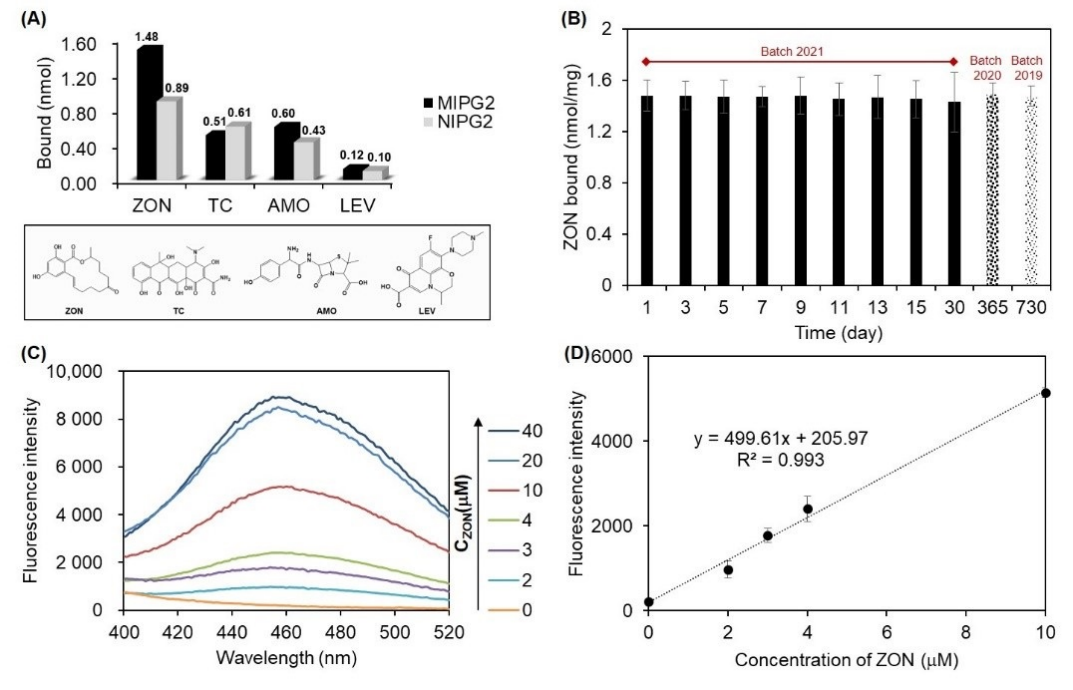
(**A**) Selectivity test of 1 mg MIPG2 and NIPG2 towards ZON and three structurally similar reagents tetracycline (TC), amoxicillin (AMO), and levofloxacin (LEV) at 2.5 μM, respectively. The chemical structure of three structurally similar reagents are given below. (**B**) Detection record of 1 mg MIPG2 (of the same batch) towards 2.5 μM ZON during 30 days after each template extraction, as well as 1 mg MIPG2 (of a different batch) towards 2.5 μM ZON in 1 and 2 years, respectively. (**C**) Analyte-dependent fluorescence emission spectra of MIPG2 with respect to ZON ranging from 0 to 40 μM in corn juice. (**D**) Lear fitting plot of fluorescence intensity of MIPG2 towards ZON in corn juice. Corn juice tests were performed on glass substrates coated with MIPG2.

In order to study the anti-interference ability of the MIPG2, the structurally similar reagents tetracycline (TC), amoxicillin (AMO), and levofloxacin (LEV) were used for the selectivity test. As shown in Figure 2A, MIPG2 exhibited lower binding towards other structural analogs with respect to ZON, demonstrating a great potential in anti-interference detecting. On the other hand, the NIPG2 shows a high cross-reactivity towards TC and AMO. Therefore, our MIPG2 is promising to detect ZON in real samples. Furthermore, the colloidal stability and batch-to-batch reproducibility were investigated prior to the real sample tests. As shown in Figure 2B, when 1 mg MIPG2 (of the same batch) was used to detect 2.5 μM ZON repeatedly after each template extraction, the binding amount was almost constant during one month, indicating its reversibility property. Meanwhile, 1 mg MIPG2 (of a different batch) was employed to recognize 2.5 μM ZON in 1 and 2 years, the amount of ZON bound remained the same, showing its excellent stability and reproducibility for sensor development.

It is well-known that ZON is a mycotoxin produced by several species of Fusarium that may contaminate cereals, in particular corn, barley, oats, wheat, and sorghum. Thus, ZON contamination can cause toxic effects in humans and animals, including nephrotoxic, neurotoxic, carcinogenic, estrogenic, and immunosuppressive effects [25,26]. Since MIPG2 exhibits good specificity and selectivity for ZON, they were then adhered on a glass substrate in order to work as a fluorescent sensor for ZON detection in real samples. Herein, we bought some corn juice as ZON-containing practical samples, and diluted it 4 times with ACN for testing. Using the standard recovery method, ZON at concentrations ranging from 0 to 40 μM were spiked in the corn juice. According to the results of ZON detection in real samples (Figure 2C,D), our MIPG2-based optical sensor may detect ZON at the linear concentration range of 0–10 μM with a limit of detection (LOD) of 1.6 μM (calculated according to the equation: D = 3σ/k, where σ is the relative standard deviation of the blank sample, k is the slope of the calibration line), showing great potential for mycotoxin detection in food testing. Although, biological molecular recognition elements exhibit lower LOD, such as the LOD of antibody-based sensors for ZON is at the nM level [30,31], and aptamer-based sensors can detect ZON at the pM level [32,33], such biosensors are expensive and have a short service life. Compared with the chromatography method which exhibited a LOD in the nM range [5], our MIPG_ZON-based fluorescent sensor is more convenient for commercial applications. Inspired by recent quantum dots-incorporated MIPG [4], our MIPG_ZON promises to be smaller and effective, and the optimization could be established on this protocol.

### 2.4. Synthesis and Binding Characterization of MIPG_GA

**Figure 3:**
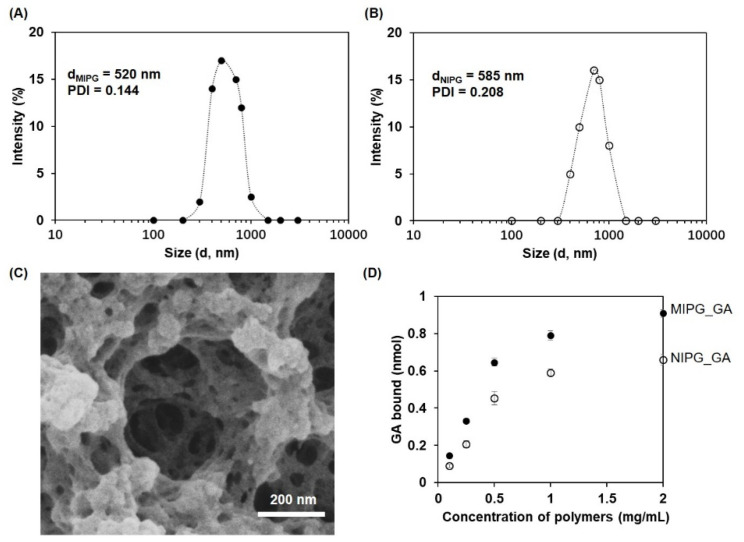
Dynamic light scattering measurements of 0.5 mg/mL (**A**) MIPG_GA and (**B**) NIPG_GA in H_2_O (mean value of two experiments). (**C**) SEM image of MIPG_GA. (**D**) Equilibrium binding isotherm for 1 mM GA on MIPG_GA and NIPG_GA at the concentrations ranging from 0.1 to 2 mg/mL in water. Data are means of three independent experiments. The error bars represent standard deviations.

The aim of this part is to synthesize a fluorescent MIPG_GA, which is capable of specifically binding towards GA (an epitope of hyaluronic acid which is usually overexpressed on cancer cells) for cell imaging. To achieve this goal, the particle size of MIPG_GA must be controlled at the nanometer scale, and the MIPG must exhibit good compatibility with water. Hence, our MIPG_GA was generated using AAB and MAM as functional monomers to create specific binding sites (Scheme 1). According to our intensive research [28,29,34], AAB is able to strongly interact with the carboxylic group and MAM is a co-monomer that establishes a hydrogen interaction with the target molecule. As shown in Figure 3A,B, the hydrodynamic size of MIPG_GA and NIPG_GA was found to be 520 and 585 nm, respectively. The SEM image (Figure 3C) shows the three-dimensional nanofiber structure of the hydrogel (FTIR analysis in Figure S5). Using these polymers to evaluate the binding performance towards GA, different concentrations of the polymers ranging from 0.1 to 2 mg/mL were incubated with 1 mM GA. After the subtraction of unbound GA from the total amount of the analyte added by the well-established Dubois method, the amount of bound GA was determined for each concentration of polymers (Figure 3D). Although the NIPG_GA bound significant amount of analyte was due to the presence of AB, which might be largely distributed on the polymer surface and very accessible to GA. It was found that MIPG_GA bound specifically to GA as the binding to NIPG_GA was lower.

### 2.5. Application of MIPG_GA-Based Fluorescent Probe for Cell Imaging

**Figure 4:**
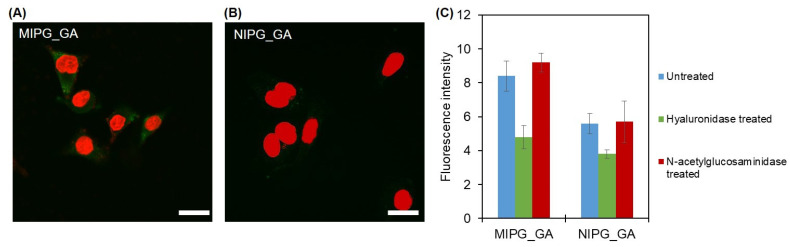
Confocal microscope images of fixed Hela cells incubated MIPG_GA (**A**) and NIPG_GA (**B**), that exhibit green fluorescence under proper filter sets of confocal microscopy. Nuclei stained with PI (red). Scale bar: 50 μm. (**C**) Summarized histogram results showing the binding performance of polymers towards untreated, hyaluronidase treated, and N-acetylglucosaminidase treated Hela cells. Data are means of three independent experiments. The error bars represent standard deviations.

The objective of this part is to observe the interaction between fluorescent polymers and Hela cells before or after the enzyme treatment, in order to demonstrate the potential of our MIPG_GA to detect cancer cells in clinical uses. The images shown in Figure 4A,B correspond to the fluorescence intensity analysis for Hela cells incubated with MIPG_GA or NIPG_GA, respectively, indicating that the MIPG_GA specifically bound towards the epitope of hyaluronic acid on Hela cells. Our fluorescent probe is more convenient for commercializing due to its excellent stability, though less sensitive with respect to the hyaluronic acid-binding protein (HABP, Figure S6) [35,36,37]. Moreover, compared with nano-sized MIP containing quantum dots [28,29], our MIPG_GA is easier to fabricate using a broad-spectrum cross-linker FAM-based monomer for signaling, showing the universal utility of this strategy.

Furthermore, according to Figure 4C, the MIPG_GA obtained from fluorescence microscope images exhibited more binding towards Hela cells with respect to the NIPG_GA. Particularly, hyaluronidase effectively removes GA on the surface of Hela cells by breaking down hyaluronic acids. This is why the fluorescence intensity for hyaluronidase treated Hela cells became lower than the untreated cells. On the other hand, N-acetylglucosaminidase plays a role to hydrolyze the non-reducing terminal of N-acetyl-D-glucosamine, whereby more GA is obtained. Thus, the fluorescence intensity of N-acetylglucosaminidase treated Hela cells increased. In addition, the MIPG_GA exhibited almost the same cell imaging fluorescence intensity in 2 years, indicating its excellent stability.

## 3. Conclusions

In summary, a MIP-based fluorescent sensor for ZON detection was successfully prepared and applied for tracing ZON in real samples. In parallel, a MIP-based fluorescent probe was fabricated and directly used for cell imaging. These two examples unfold the beauty of molecular imprinting technology in front of our eyes, indicating the perspectives and versatility of this technique for designing and fabricating fluorescent sensors towards commercial applications. The biggest gain in this work is the discovery of the road towards simple and real-world applications for the MIPGs, showing a great potential of this kind of fluorescent sensors in the detection of analyte of interest in food and biological samples.

## 4. Materials and Methods

### 4.1. Reagents

The (4-acrylamidophenyl)(amino)methaniminium acetate (AAB) was synthesized as previously described [34]. The 4-vinylpyridine (4-VPY), methacrylamide (MAM), ethylene glycol dimethacrylate (EGDMA), azo-bis-dimethylvaleronitrile (ABDV), azobisisobutyronitrile (AIBN), zearalanone (ZAN), zearalenone (ZON), hyaluronidase, N-acetylglucosaminidase, hyaluronic acid-binding protein (HABP), biotin, streptavidin, and fluorescein-5-isothiocyanate (FITC) was purchased from Sigma-Aldrich (Shanghai, China). Fluorescein dimethacrylate (PolyFluor^®^ 511) was obtained from Polysciences (Hirschberg an der Bergstrasse, Germany). Glucuronic acid (GA), tetracycline (TC), amoxicillin (AMO), and levofloxacin (LEV) came from Sinopharm Chemical Reagent Co., Ltd. (Shanghai, China). All the solvents used in the experiments as well as acids and bases are of analytical grade and anhydrous for acetonitrile (ACN) and dimethylsulfoxide (DMSO). The materials for cells culturing are: Phosphate buffer saline (PBS), penicillin/streptomycin, high glucose Dulbecco’s Modified Eagle’s Medium (DMEM) supplied with L-glutamine, fetal bovine serum (FBS), trypsin, ethylenediaminetetraacetic acid, and were obtained from Thermo Fisher Scientific (Shanghai, China).

### 4.2. Synthesis of MIPGs_ZON and MIPG_GA

**MIPGs_ZON** were synthesized in the presence of the ZAN using different functional monomer ratios. The 4-VPY was distilled under reduced pressure before use. First of all, 10 mg (0.03 mmol) ZAN, 6.4 or 12.7 or 19.2 μL (0.06, 0.12, 0.18 mmol) 4-VPY, and 113.1 μL (0.60 mmol) EGDMA was mixed in 400 μL of ACN. After sonication for 5 min, the initiator ABDV (1 mol% with respect to the number of double bonds) was added. Then, the prepolymerization mixture was purged with nitrogen for 5 min and placed in a water bath maintained at 40 °C overnight. The non-imprinted polymeric hydrogels (NIPGs) are synthesized in the same way but without the template. For template extraction, the polymers were washed 3 times with methanol/acetic acid (7/3), once with methanol, 3 times methanol containing 2.8% ammonia, and 3 times with methanol for 1 h at room temperature. The final polymers were obtained after drying in a vacuum overnight.

**MIPG_GA** was synthesized by mixing 4.85 mg (0.025 mmol) GA, 4.73 mg (0.025 mmol) AAB (1 h pre-incubation with GA), 6.38 mg (0.075 mmol) MAM, and 234 mg (0.5 mmol) PolyFluor^®^ 511 in 1903 μL DMSO. Afterwards, 1.01 mg (0.0062 mmol) AIBN was added and before nitrogen purging for 5 min. The mixture was then heated overnight at 50 °C. The next day, a precipitated polymer was obtained. The NIPGs were synthesized in the same way but without the template. For template extraction, the polymers are washed 3 times with 1 M hydrochloric acid, once with methanol, 3 times methanol containing 2.8% ammonia, and 3 times with methanol for 1 h at room temperature. The final polymers were obtained after drying in a vacuum overnight.

The sizes of synthesized polymers were measured by dynamic light scattering (DLS) analysis on a Zetasizer NanoZS at 25 °C, in ACN for MIPs_ZON and in H_2_O for MIP_GA. Scanning electron microscopy (SEM) imaging was carried out on a Quanta FEG 250 scanning electron microscope (FEI Europe), by spraying gold. Prior to the binding studies, the dried MIPGs were added into a dialysis bag for checking whether there was any leakage of the template one day later. If there is any leakage, a second round of washing will be carried out. Afterwards, the MIPG2_ZON and MIPG_GA were analyzed by Fourier transform infrared spectrometer (FTIR), respectively. In addition, 5 mg/mL MIPG2_ZON in ACN and 5 mg/mL MIPG_GA in DMSO images were taken, in order to prove the gel format.

### 4.3. Equilibrium Binding Studies

For **MIPGs_ZON,** the equilibrium binding experiments with ZON were performed in anhydrous acetonitrile. The particles of 10 mg MIPGs_ZON and NIPGs_ZON were suspended in 1 mL ACN with intensive sonication (ultrasonic power: 80 W, frequency: 40 KHz, 20 min), in order to work as stock solutions, respectively. Then, polymer concentrations at 0.1, 0.25, 0.5, 1, 2, 5 mg/mL were incubated with 2.5 µM ZON in 1 mL ACN overnight. The samples were then centrifuged at 17,500 rpm for 20 min and a 700 μL aliquot of the supernatant was taken for unbound ZON determination. Then, the amount of bound ZON was calculated by subtracting the amount of unbound from the total. A calibration curve of ZON (Ex = 280 nm, Em = 455 nm, slit: 5) was obtained on F-7000 spectrofluorometer (Hitachi High-Technologies, Tokyo, Japan), as shown in Figure S1. To investigate the binding capacity, 1 mg of polymers were incubated with ZON varying from 0.5 to 10 μM in 1 mL ACN, with results plotted with the Langmuir-type isotherm equation. The selectivity study was performed by testing the binding of 1 mg polymers towards 2.5 μM ZON, and three structurally similar reagents: Tetracycline (TC), amoxicillin (AMO), and levofloxacin (LEV).

For **MIPG_GA,** the recognition properties were evaluated using the method of Dubois [28]. Dubois’s method is a colorimetric method widely used to determine monosaccharide based on the preconversion of sugars into furfural derivatives upon heating with strong acids, followed by the formation of a colored complex with phenol. Prior to the binding assay, a calibration curve was generated with standard solutions of GA at concentrations ranging from 0.1 to 5 mM in water (Figure S2). Afterwards, the polymers at concentrations varying from 0.1 to 2 mg/mL were incubated with 1 mM GA in 1 mL water overnight. The samples were then centrifuged at 17,500 rpm for 1 h, in order to provide the supernatant for GA determination by Dubois’s method.

### 4.4. Application of MIPG_ZON-Based Fluorescent Sensor for Real Sample Tests

Initially, 100 mg MIPG2_GA and NIPG2_GA were mixed with 480 mg poly (vinyl alcohol) in water and smeared on the glass plates, respectively. Herein, poly (vinyl alcohol) were used as an adhesion agent due to its excellent transparent feature, exhibiting no interference to ZON excitation light. After 30 min of shaking in an oven at 90 °C, the glass plates were left for 1 h at room temperature to cool down. The glass plates coated with MIPG2_ZON and NIPG2_ZON were stored in the fridge at 4 °C until use. For the real sample tests, the spiked samples were prepared using ZON at concentrations from 0–40 μM dissolved in DMSO. The prepared ZON samples were then mixed with corn juice at a ratio of 1:9 for the determination of recovery. For fluorescence intensity measurements after 1 h incubation, the Cary Eclipse fluorescence spectrophotometer (Varian, Palo Alto, CA, USA) was used.

### 4.5. Application of MIPG_GA-Based Fluorescent Probe for Cell Imaging

Cells were grown in a 5% CO_2_ incubator at 37 °C using DMEM supplemented with 10% premium FBS and antibiotics (100 units/mL penicillin and 100 μg/mL streptomycin) in cell culture dishes. For imaging studies, Hela cells were grown on cover slips and then incubated with 0.5 mg/mL polymers or 50 ng/mL FAM-HABP (synthesized in two steps, first with a biotinylated HABP, followed by incubation with streptavidin-FITC [37]) for 1 h. The cell monolayers were then washed 3 times with PBS (pH 7.4) and immediately measured by a Leica TSC SP5 confocal scanning laser microscopy (CSLM) system (Ex = 480, Em = 515).

## Figures and Tables

**Table 1:** Composition of ZAN imprinted polymers synthesized in 400 μL anhydrous ACN and their corresponding sizes.

Polymer	ZAN (mmol)	4-VPY (mmol)	EGDMA (mmol)	ABDV (mg)	Size (nm)	PDI
MIPG1	0.03	0.06	0.6	3.1	345 ± 5	0.163
NIPG1	-	0.06	0.6	3.1	455 ± 16	0.160
MIPG2	0.03	0.12	0.6	3.2	443 ± 11	0.137
NIPG2	-	0.12	0.6	3.2	483 ± 6	0.199
MIPG3	0.03	0.18	0.6	3.4	468 ± 12	0.156
NIPG3	-	0.18	0.6	3.4	503 ± 9	0.085

## Supplementary Materials

The following are available online at https://www.mdpi.com/article/10.3390/gels7020067/s1. Figure S1: Fluorescence calibration curve of ZON in ACN (Ex = 280 nm, Em = 455 nm, slit: 5 nm); Figure S2: Calibration curve of glucuronic acid in water (quantified by Dubois’s method with absorbance read at 490 nm). Figure S3: Equilibrium binding isotherms for 2.5 μM ZON on ZON-imprinted polymers (MIP) and NIP at concentrations ranging from 0.25 to 2 mg/mL in 1 mL ACN. MIP here were synthesized using the similar protocol for MIPG2, in the presence of ZON. Data are means from three independent experiments. The error bars represent standard deviations. Figure S4: FT-IR spectra of MIPG2_ZON with solution image insert, where some special peaks were highlighted. Figure S5: FT-IR spectra of MIPG_GA with solution image insert, where some special peaks were highlighted. Figure S6: Confocal microscope images of fixed Hela cells incubated MIPG_GA (A) and FAM-HABP (B), that exhibit green fluorescence under proper filter sets of confocal microscopy. Nuclei stained with PI (red). Scale bar: 50 μm.
