# Supplementary material for: Typical Fluorescent Sensors Exploiting Molecularly Imprinted Hydrogels for Environmentally and Medicinally Important Analytes Detection
Source: Gels. 2021 Jun 8;7(2):67. doi: 10.3390/gels7020067 (PMC8293044; doi:10.3390/gels7020067)
Supplement: Supplementary file 1 [file gels-07-00067-s001.zip › gels-1230577-supplementary.pdf]

Supplementary information

Typical fluorescent sensors exploiting molecularly imprinted hydrogels for environmentally and medicinally important analytes detection

Lihua Zou [1#], Rong Ding [2#], Xiaolei Li [2], Haohan Miao [3], Jingjing Xu [1,2,*] and Guoqing Pan [3,*]

[1] Center for Molecular Recognition and Biosensing, School of Life Sciences, Shanghai University, Shanghai 200444, P. R. China

[2] Sino-European School of Technology of Shanghai University, Shanghai University, CN-200444 Shanghai, P. R. China

[3] Institute for Advanced Materials, School of Materials Science and Engineering, Jiangsu University, Zhenjiang 212013, Jiangsu, China

[#] Both contributed equally to the work.

* Corresponding authors: jingjing_xu@shu.edu.cn (J. Xu); panguoqing@ujs.edu.cn (G. Pan).

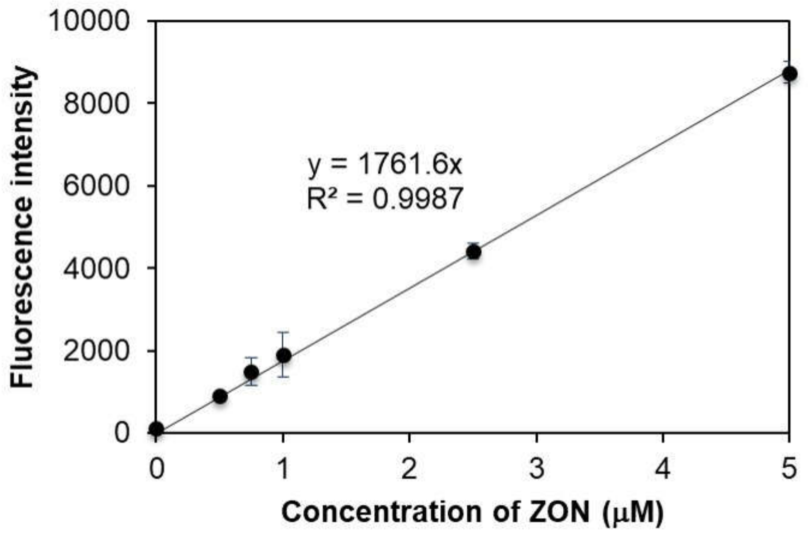

Figure S1. Fluorescence calibration curve of ZON in ACN (Ex = 280 nm, Em = 455 nm, slit: 5 nm).

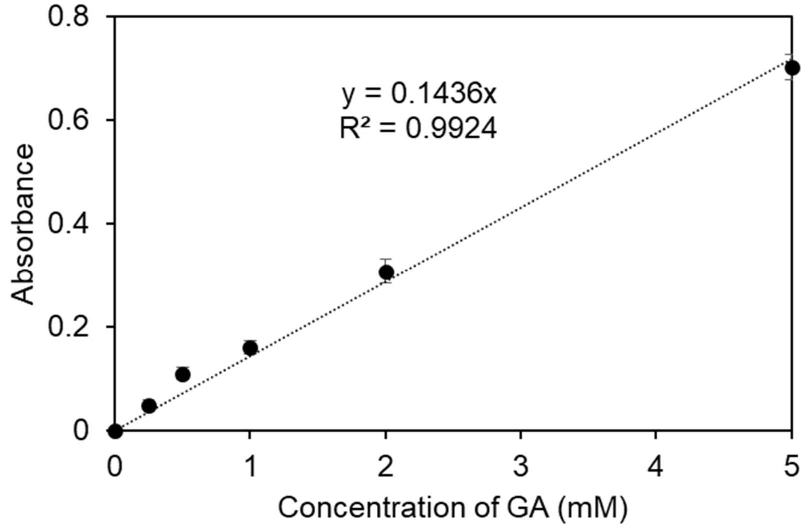

Figure S2. Calibration curve of glucuronic acid in water (Quantified by the method of Dubois with absorbance read at 490 nm).

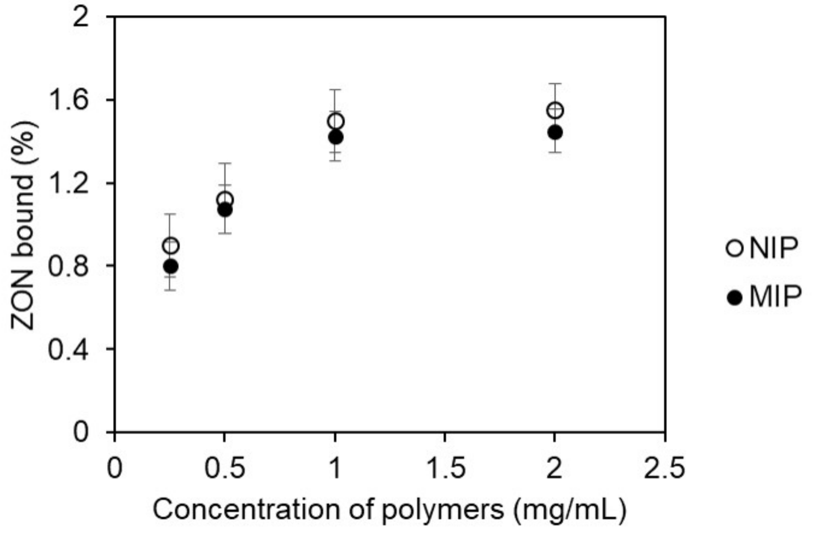

Figure S3. Equilibrium binding isotherms for 2.5 μM ZON on ZON-imprinted polymers (MIP) and NIP at concentrations ranging from 0.25 to 2 mg/mL in 1 mL ACN. MIP here were synthesized using the similar protocol for MIPG2, in the presence of ZON. Data are means from three independent experiments. The error bars represent standard deviations.

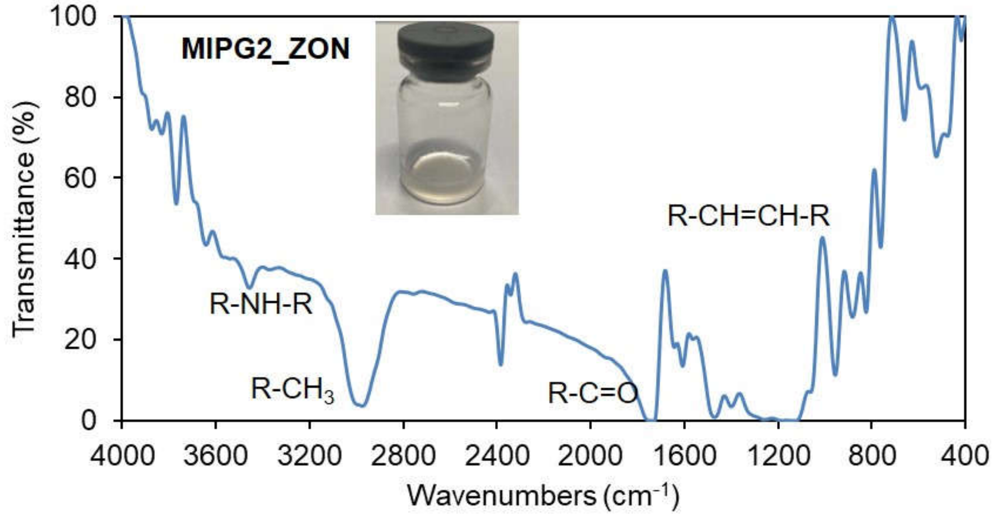

Figure S4. FT-IR spectra of MIPG2_ZON with solution image insert, where some special peaks were highlighted.

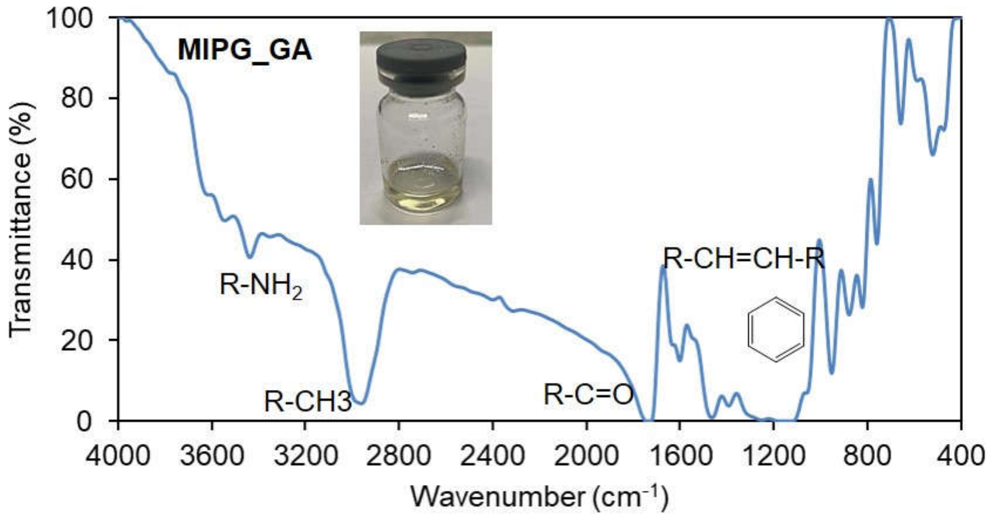

Figure S5. FT-IR spectra of MIPG_GA with solution image insert, where some special peaks were highlighted.

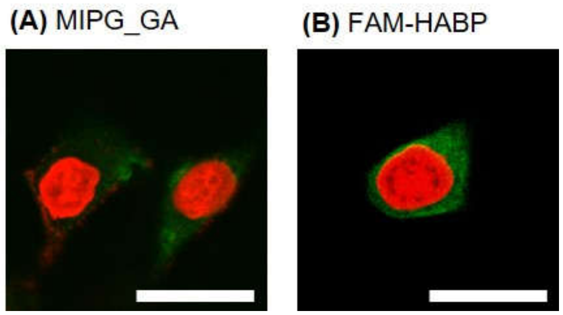

Figure S6. Confocal microscope images of fixed Hela cells incubated MIPG_GA (A) and FAM-HABP (B), that exhibit green fluorescence under proper filter sets of confocal microscopy. Nuclei stained with PI (red). Scale bar: 50 μm.
